# Use of low-dose varenicline in hospitalized smokers: A case series low-dose varenicline in hospitalized smokers

**DOI:** 10.1177/2050313X251338637

**Published:** 2025-05-05

**Authors:** Rachael L. Joyner, Remi E. Phillips, Farid A. Manshaii, James M. Davis

**Affiliations:** 1 Duke Center for Smoking Cessation, Duke University School of Medicine, Durham, NC, USA; 2 Duke Cancer Institute, Duke University, Durham, NC, USA; 3 Department of Psychology and Neuroscience, University of North Carolina, Chapel Hill, NC, USA; 4 Department of Psychiatry and Biobehavioral Sciences, University of California, Los Angeles, Los Angeles, CA, USA; 5 Department of Medicine, Duke University School of Medicine, Durham, NC, USA

**Keywords:** smoking cessation, varenicline, hospitalization

## Abstract

Nicotine replacement therapy is the primary medication used to treat tobacco dependence in hospitalized patients. Recent evidence shows that varenicline is more effective than nicotine replacement, but varenicline side effects limit its use in the hospital. Low-dose varenicline has shown similar efficacy as standard dose, but has fewer side effects and thus may be useful in hospitalized patients. We assessed the effectiveness and tolerability of low-dose varenicline (1 mg each morning or 0.5 mg twice daily; patient choice) in hospitalized adult daily smokers between July 2022 and March 2023. The primary outcome was self-reported 7-day smoking abstinence at the first outpatient visit after hospitalization. Secondary outcomes included smoking reduction and medication tolerability. Of the 15 patients assessed, 8 (53.3%) reported 7-day smoking abstinence at their first post-discharge outpatient visit (mean of 14.9 days after discharge). Mean cigarettes per day decreased from 22.0 (SD 17.4) prior to hospitalization to 3.7 (SD 6.0) after hospitalization. Low-dose varenicline was well tolerated, with 80% (12) patients reporting no adverse events and 20% (3) patients reporting mild side effects not requiring a change in medication or dose. Study outcomes support the possibility that low-dose varenicline might be an effective and well-tolerated treatment for hospitalized smokers. Results from this case series are limited by the small sample size and lack of a control arm. Findings suggest that a randomized controlled trial may be warranted to assess the efficacy and tolerability of low-dose varenicline in hospitalized patients.

## Background

Smoking is the leading cause of preventable illness and death in the United States, with ~480,000 deaths annually, and 16 million who suffer from a smoking-related illness.^1^ Smoking is known to cause 12 types of cancer, heart disease, vascular disease, lung diseases, and diabetes, and reduces life expectancy by ~10 years.^2^

There are currently seven FDA-approved smoking cessation medications, including five forms of nicotine replacement therapy (NRT; nicotine gum, lozenge, transdermal patch, nasal spray, and inhaler) and two oral medications (bupropion and varenicline).^3^ A 2013 Cochrane meta-analysis of 267 randomized trials (*N* = 101,804) showed that all medications were more efficacious than placebo: NRT (odds ratio (OR) 1.84), bupropion (OR 1.84), varenicline (OR 2.88); and that varenicline was more efficacious than NRT (OR 1.57; 95% confidence interval (CI) 1.29–1.91).^4^ It is now well established that varenicline is the most efficacious single smoking cessation pharmacotherapy.^5^

There are substantial benefits to treating smokers while hospitalized. Many hospitalizations are caused by smoking-related illnesses, including chronic obstructive pulmonary disease (COPD),^6^ cardiovascular disease,^7^ and multiple cancers.^8^ Moreover, hospitalization may result in a “teachable moment” in which patients are more willing to attempt smoking cessation.^9,10^ In addition, since the Joint Commission on Accreditation of Healthcare Organizations hospital-based smoking bans,^11^ smokers undergo enforced smoking abstinence while hospitalized.^12^

A 2012 Cochrane group meta-analysis of 50 trials on hospital-based smoking cessation treatments showed that the addition of nicotine replacement to intensive counseling increased smoking abstinence compared to intensive counseling alone (relative risk (RR) 1.54). The addition of varenicline to intensive counseling did not increase abstinence (RR 1.28; 95% CI 0.95–1.74), but only two trials were available^13,14^ and significance was limited by small sample size and heterogeneity. In a more recent study (*N* = 392) of hospitalized smokers, biochemically verified smoking abstinence for varenicline plus counseling was 29.2% compared to counseling alone, 18.8% (OR 1.78; 95% CI 1.10–2.86).^15^ In this study, participants using varenicline showed ~10-fold increase in nausea (16.3%) compared to controls (1.5%). This most recent study suggests that although varenicline may be quite efficacious for hospital-based smoking cessation, its utility may be limited by a relatively high incidence of side effects.

Varenicline-associated side effects have been thoroughly explored. In 2009, the FDA issued a Black Box warning for varenicline due to concerns of psychiatric symptoms and suicidality.^16^ In 2016, the Evaluating Adverse Events in a Global Smoking Cessation Study trial (*n* = 8144) showed that varenicline did not lead to an increased incidence of moderate-to-severe neuropsychiatric events or suicidality,^17^ and the FDA removed the Black Box warning.^18^ Still, Pfizer reported varenicline caused nausea in 30% and insomnia in 18% of patients.^19^ Other studies have also found that varenicline has a relatively high incidence of side effects—especially nausea and insomnia.^20,21^ Medication side effects have been recognized as a major problem for hospitalized patients, and wide-scale efforts are being undertaken to decrease medication side effects in hospitalized patients.^22^

Low-dose varenicline appears to show similar efficacy but with much better tolerability. During the Phase-2 FDA approval study conducted by Pfizer, 12-week post-quit smoking abstinence rates for standard-dose varenicline (1 mg twice daily) were 49.4% and low-dose varenicline (0.5 mg twice daily) were 44.0%—both higher than placebo (11.6%).^23^ Importantly, nausea occurred in 34.0% of standard-dose varenicline, but only 16.3% for low-dose varenicline—very similar to 14.9% for placebo.^23^ Similarly, a 2017 study found 12-month smoking abstinence of 46.5% for standard-dose varenicline (1 mg twice daily) and 46.4% for low-dose varenicline (0.5 mg twice daily; OR 1.0).^24^ Rates of nausea with standard-dose varenicline were numerically higher (19.3%) compared to low-dose varenicline (12.1%).^24^ Thus, the evidence appears to show that low-dose varenicline has similar efficacy, but a lower incidence of side effects, especially nausea, compared to standard-dose varenicline. These findings suggest that low-dose varenicline may be a reasonable treatment for hospitalized smokers.

## Case series

This case series was designed to assess smoking abstinence rates, smoking reduction, and side effects in smokers who used low-dose varenicline during hospitalization. The case series was observational and followed a natural course of treatment. The case series did not posit a priori hypotheses with statistical benchmarks, but it was designed with the expectation that low-dose varenicline might be relatively effective and well tolerated in hospitalized patients.

This study was conducted at Duke University Hospital, within a large university health system in Durham, North Carolina, USA. The study was reviewed by the Duke University Health System Institutional Review Board and was determined to be exempt because it contained no intervention aside from routine medication treatment provided by Duke Smoking Cessation Program providers, who routinely prescribe low-dose varenicline. Study participants were Duke Smoking Cessation Program patients hospitalized between July 2022 and March 2023.

Inclusion criteria were that patients had to be hospitalized adults (⩾18 years of age), smoked cigarettes daily prior to admission, started on low-dose varenicline while in the hospital, and attended at least one outpatient follow-up with the Duke Smoking Cessation Program after discharge. Follow-up after discharge was necessary to assess smoking abstinence. As an observational study of real-world treatment, the study did not have exclusion criteria. However, program clinicians typically avoid prescribing varenicline to certain patients, including those with uncontrolled psychiatric symptoms, nausea/vomiting, or insomnia.

Patients were seen by a medical provider who provided them with a minimum of 20 min of smoking cessation counseling. All patients were started on low-dose varenicline while in the hospital and were allowed to choose between varenicline 1 mg once daily or 0.5 mg twice daily. An explanation was provided that 0.5 mg twice daily may be less likely to cause nausea, while varenicline 1 mg once daily may be less likely to cause sleep problems. The reason why either dose was deemed to be acceptable is that the half-life of varenicline is 24 h.^19^ This means that with once daily dosing, the early morning serum levels should be roughly 50% of the maximum, and serum levels during sleep should be lower, potentially reducing sleep problems. We did not use up-titration because low-dose varenicline without up-titration was tolerated well in the Pfizer Phase 2 dosing study.^23^

We collected baseline demographics and smoking history on all program patients and recorded them in the electronic health record. The primary outcome was self-reported 7-day point-prevalence smoking abstinence assessed at the first outpatient post-hospitalization smoking cessation follow-up visit. Biochemical verification of smoking status was not performed because follow-up visits were conducted via telehealth due to the wide geographic distribution of our patient population. Secondary outcomes included a change in daily cigarettes smoked per day and varenicline tolerability assessed at each visit via an open-ended question and also through inquiry on a list of common varenicline side effects (nausea, vomiting, vivid dreams, and insomnia). Most analyses were descriptive and used Microsoft Excel 2023.

## Results

A total of 15 patients met the criteria for the case series, mean patient age was 54 years, 6 (40%) were female, 6 (40%) were Black, and 9 (60%) were White. Prior to hospitalization, patients smoked an average of 22.0 cigarettes/day (Table 1). Of 15 patients in the case series, 12 (80%) chose to use varenicline 1 mg once daily and 3 (20%) chose varenicline 0.5 mg twice daily.

**Table 1.** Demographics and smoking history.

Average, age	54 years (SD = 12)
Gender	Male (*n* = 9, 60%), female (*n* = 6, 40%)
Race	Black (*n* = 6, 40%), Caucasian (*n* = 8, 53.3%), Native Indian/Pacific Islander (*n* = 1, 6.7%)
Insurance	Commercial insurance (*n* = 6, 40%), Medicaid (*n* = 6, 40%), Medicare (*n* = 3, 20%)
Average lifetime cigarettes smoked per day	27.1 (SD 13.3)
Average age of smoking initiation	15.6 years (SD 2.9)
Average cigarettes smoked per day prior to hospitalization	22.0 (SD 17.4)

Primary admitting diagnoses included myocardial infarction, with coronary artery disease (CAD), chronic obstructive pulmonary disease (COPD) exacerbation, osteomyelitis, lung cancer, congestive heart failure (CHF), gastrointestinal bleeding, abdominal aortic aneurysm (AAA), parapneumonic effusion, sepsis, cardiomyopathy, lower limb ischemia, hypotension, deep vein thrombosis (DVT), pulmonary embolism (PE), stroke, transischemic attack (TIA) and ventricular arrhythmia. The mean length of stay was 14.4 days (SD 12.4). Of the 15 patients in the case series, 14 had a history of smoking-related illness, and nine had a diagnosis of mental illness including anxiety, depression, bipolar disorder, post-traumatic stress disorder (PTSD) and/or substance abuse (Table 2).

**Table 2.** Smoking-related illness, mental illness, substance abuse, and comorbidities.

Smoking-related illness		Mental illness, substance abuse		Multiple comorbidities
COPD	*n* = 7 (50%)	Anxiety	*n* = 5 (55.6%)	Seven patients had two major comorbidities
Asthma	*n* = 1 (7.1%)	Depression	*n* = 5 (55.6%)	Four patients had three or more major comorbidities
CAD	*n* = 7 (50%)	Bipolar	*n* = 1 (11.1%)	
Stroke/TIA	*n* = 1 (7.1%)	PTSD	*n* = 1 (11.1%)	
Vascular disease	*n* = 3 (21.4%)	Substance abuse	*n* = 2 (22.2%)	
Diabetes	*n* = 7 (50%)			
Cancer	*n* = 2 (14.3%)			
Hypertension	*n* = 11 (78.6%)			
AAA	*n* = 3 (21.4%)			
DVT/PE	*n* = 1 (7.1%)			

AAA: abdominal aortic aneurism; CAD: coronary artery disease; COPD: chronic obstructive pulmonary disease; DVT: deep vein thrombosis; PE: pulmonary embolism; PTSD: post-traumatic stress disorder; TIA: transischemic attack.

Of the 15 patients in the case series, 8 (53.3%) demonstrated self-reported 7-day point prevalence of smoking abstinence at the first post-hospitalization follow-up visit. Among the seven patients who were still smoking, all reported >50% smoking reduction compared to baseline smoking. The average cigarettes per day reduced from 22.0 (SD 17.4) prior to admission to 3.7 (SD 6.0) at the post-hospitalization follow-up visit. The mean time to the first outpatient follow-up was 14.9 days (SD 12.4).

By dosing regimen, the self-reported 7-day smoking abstinence rate was 70.0% (7/10) for those taking 1 mg once daily and 66.7% (2/3) for those taking 0.5 mg twice daily. The sample was too small for meaningful statistical comparison (χ^2^ = 0.00, *p* = 1.00). The mean age of abstinent participants was 56.0 years, while the mean age of non-abstinent participants was 48.25 years (nonsignificant). Due to the small sample size, significance testing was not conducted to compare abstinence rates by gender, race, or ethnicity.

Of the 15 patients in the case series, 12 (80%) reported no side effects related to varenicline, while 3 (20%) reported mild side effects related to varenicline (rated 1–2 out of 7-point Likert scale; 1–2 = mild, 3–5 = moderate, 6–7 = severe). Of these, one had nausea, two had vivid dreams, and none requested to stop taking varenicline or reduce varenicline dosing. There were no reports of psychiatric symptoms (depression, anxiety, and impulsiveness) and no reports of vomiting, insomnia, or nightmares). Because the group with side effects was so small (3) it was not possible to identify a relationship between side effects and underlying physical or psychiatric comorbidity.

## Discussion

This small case series provides preliminary data showing that low-dose varenicline may be effective and well tolerated for hospitalized smokers. The study suggests an alternative to using the standard dose of varenicline assessed in previous studies.^13–15^ The fact that most patients in the case series were hospitalized for significant health conditions, with many suffering from mental health or addiction, suggests the possibility that low-dose varenicline may be effective and well tolerated in hospitalized patients with high comorbidity. A key component of this case series is that all patients received intensive counseling (a minimum of 20 min), and all were seen after hospitalization at an outpatient follow-up visit. In hospitalized smokers, intensive counseling and outpatient follow-up are both associated with higher rates of smoking abstinence.^12^ Because only three patients utilized varenicline 0.5 mg twice daily, there was insufficient sample size to compare the effects of 0.5 mg twice daily to 1 mg once daily dosing.

A notable feature of this study design is that patients were allowed to choose between two low-dose varenicline regimens (1 mg once daily or 0.5 mg twice daily). The majority (80%) opted for 1 mg daily. While we did not have the power to statistically compare the efficacy between these regimens, both doses appeared to be moderately effective and well tolerated. Further research could explore if differences exist between once-daily versus twice-daily low-dose varenicline regimens.

Study limitations include a small sample size and the absence of a control group, which limits the ability to establish causal relationships or compare outcomes to standard treatments. As a case series, this study does not provide a high degree of certainty regarding outcomes but does provide preliminary evidence to support further investigation. The presence of behavioral treatment (intensive counseling and outpatient follow-up) likely contributed to smoking abstinence, thus treatment effects likely resulted from a combination of medications and counseling and the therapeutic contribution of each is unknown. A significant limitation of this study was the relatively short follow-up period (mean 14.9 days), which is less predictive of long-term smoking abstinence than assessment at later time points. Although expert consensus has identified 6- and 12-month follow-ups as gold standards in the field,^25^ evidence also supports the more limited prognostic value of short-term abstinence. Two studies show that smokers who achieve smoking abstinence for 2 weeks are approximately four times as likely to achieve 6-month abstinence compared to those who did not achieve 2-week smoking abstinence.^26,27^ Thus, while short-term abstinence outcomes should not be conflated with longer-term outcomes, they offer meaningful insight into treatment efficacy. Future studies on low-dose varenicline should aim to include a longer follow-up period to assess smoking abstinence. Another limitation of this study is that smoking abstinence was assessed by self-report without biochemical verification. The reason for this is that Duke University Hospital provides care for patients across a large catchment area and follow-up visits are normally conducted via telehealth, making biochemical verification (e.g. urinary cotinine levels) impractical. While this approach reflects routine clinical practice for telehealth visits, self-reported abstinence is less accurate than biochemically verified abstinence. That said, prior research has shown that self-report is generally accurate, with a meta-analysis reporting 87.5% sensitivity and 89.2% specificity relative to biochemical measures.^28^ Future studies would benefit from incorporating biochemical verification to enhance the accuracy of abstinence testing. In addition, the study did not incorporate open-ended patient surveys to assess subjective experiences with varenicline therapy.

One important question that was not addressed by this study is: *under what conditions should a clinician initiate low-dose varenicline in hospitalized patients?* Although existing literature describes the incidence of side effects—most commonly nausea and sleep disturbances^29^—real-world prescribing practices and motivations for choosing a particular therapy over another may differ substantially between providers and were not assessed in this study. An evaluation of clinician decision-making regarding the use of low-dose varenicline in hospital settings would be a valuable contribution to the field.

Finally, as the case series was conducted at a single university hospital in the southeastern US findings may not be generalizable to other hospitals or geographic regions. In the face of these limitations common in case series, the key findings in this study—reductions in smoking and favorable tolerability—suggest that low-dose varenicline may be a viable treatment option for hospitalized smokers. Importantly, findings provide sufficient evidence to conduct a comparative trial to assess the efficacy and tolerability of low-dose varenicline in hospitalized patients.

## Conclusion

Low-dose varenicline used during hospitalization in a small series of patients demonstrated promising tolerability and preliminary efficacy. This is an important finding because varenicline is a highly effective medication but has limited use in hospitalized patients likely due to concerns about side effects. Findings suggest that a randomized controlled trial may be warranted to assess the efficacy and tolerability of low-dose varenicline in hospitalized patients.
